# Effects of switching from intravitreal injection of aflibercept to faricimab on ocular blood flow in patients with diabetic macular edema

**DOI:** 10.1038/s41598-024-63435-8

**Published:** 2024-06-14

**Authors:** Yoshinari Saima, Harumasa Yokota, Akifumi Kushiyama, Junya Hanaguri, Akira Ohno, Koyo Takase, Ruri Sugiyama, Kimimasa Muranaka, Satoru Yamagami, Taiji Nagaoka

**Affiliations:** 1 Division of Ophthalmology, Department of Visual Sciences, Nihon University School of Medicine, Tokyo, Japan; 2 Department of Ophthalmology, Saitama Medical Center, Jichi Medical University, Saitama, Japan; 3 Department of Pharmacotherapy, Meiji Pharmaceutical University, Tokyo, Japan; 4 Tokiwadai Muranaka Eye Clinic, Tokyo, Japan; 5 Department of Ophthalmology, Asahikawa Medical University, Asahikawa, Hokkaido Japan

**Keywords:** Circulation, Medical research

## Abstract

We assessed the short-term effects of switching from intravitreal aflibercept (IVA) to intravitreal faricimab (IVF) on ocular blood flow in patients with treatment-resistant diabetic macular edema (DME). The medical records of 15 patients with DME who had received IVA injection ≥ 3 months before were retrospectively reviewed. The best-corrected visual acuity, central macular thickness (CMT) on optical coherence tomography, and mean blur rate (MBR) of all disc areas on laser speckle flowgraphy were measured before, 1 week after, and 4 weeks after IVA and IVF, respectively. The changes in visual acuity showed no significant difference after switching from IVA to IVF (P = 0.732). The mean CMT decreased significantly during the follow-up period (both P < 0.001). MBR showed no significant difference during the follow-up period (P = 0.26). However, it decreased significantly 4 weeks after IVF (P = 0.01) compared with the baseline value, but not 4 weeks after IVA (P = 0.074). A significant association was observed between decreased MBR and decreased CMT in patients who received IVF (correlation coefficient: 0.501, P = 0.005) but not in those who received IVA (P = 0.735). Thus, IVF maintained ocular blood flow reduction, although no significant differences in visual acuity and CMT changes were observed compared to IVA.

## Introduction

Diabetic macular edema (DME) is characterized by the leakage of vascular components owing to dysfunction of the retinal capillary endothelial cells and increased permeability of the vessel wall induced by hyperglycemia. Macular edema can lead to visual dysfunction, especially in cases involving the central fovea. DME is estimated to affect approximately 21 million individuals worldwide^1^. Conventional treatments for DME include the local administration of steroids, retinal laser photocoagulation, and vitrectomy. However, intravitreal injection of anti-vascular endothelial growth factor (VEGF) antibody agents has gained popularity in recent years owing to its effectiveness. Ranibizumab, aflibercept, and brolucizumab are some of the commonly used anti-VEGF antibody agents used in the treatment of DME.

Faricimab, a bispecific antibody that inhibits VEGF-A and angiopoietin-2 (Ang-2), was launched in Japan in 2022. Global Phase III clinical trials^2^ have shown that faricimab is not inferior to aflibercept in terms of its primary endpoint and that it is the first intraocular agent to achieve durability at intervals of up to 16 weeks. Anti-VEGF antibody therapy is expensive and requires re-administration of the drug with each recurrence of DME; thus, the interval between consecutive drug injections has continued to pose a problem. Faricimab is a promising drug that could reduce the treatment burden for DME.

The measurement of fundus blood flow may aid in elucidating the mechanisms underlying retinal diseases related to blood flow and vessels, such as diabetic retinopathy, and developing new treatment strategies. Doppler imaging, laser Doppler velocimetry, and laser speckle flowgraphy (LSFG) are non-invasive methods that have been used to measure fundus blood flow. LSFG measures the fundus blood flow by detecting the speckle contrast pattern produced by the interference of laser light scattered by the movement of erythrocytes in blood vessels. It is the most convenient method used in clinical practice. The changes in this contrast pattern have been used to calculate the relative blood flow in the optic nerve papilla and vessels of the retina, which is expressed as the mean blood flow velocity (MBR)^3^.

Retinal MBR and choroidal blood flow have been shown to decrease in patients with DME after ranibizumab vitreous injection^4^. A decrease in the choroidal blood has been observed in patients who did not undergo panretinal photocoagulation^5^. A decrease in retinal blood flow has also been reported in patients with DME after receiving intravitreal aflibercept (IVA), possibly due to reduced retinal artery diameter^6^. In addition, using a laser doppler flowmeter, we have previously revealed that choroidal blood flow in the foveal region was reduced in patients with DME, suggesting that the changes in ocular blood flow may be associated with the pathogenesis of DME^7^. However, to the best of our knowledge, no previous studies have compared the retinal blood flow in patients with DME before and after administering intravitreal faricimab (IVF) injection.

Therefore, this study examined the changes in the corrected visual acuity, central macular thickness (CMT), and retinal microcirculation before and after drug administration in patients with DME who switched to IVF from IVA for the treatment of recurrent edema. This study aimed to assist in examining the effects of IVF on ocular circulation in patients with DME.

## Results

Fifteen patients, comprising 10 males and five females with a mean age of 56.8 years (35–81), were included in this study. Table 1 presents the demographics at baseline (before the injection).

**Table 1:** Patients characteristics at baseline.

N = 15 patients/15 eyes	
Age (years, mean ± SD)	56.8 ± 12.9
Sex (N, %)	
Male	10 (66.6%)
Female	5 (33.3%)
HbA1c (%, mean ± SD)	7.5 ± 0.7
eGFR (mL/min/1.73 m^2^, mean ± SD)	71.3 ± 31.4

*eGFR* estimated glomerular filtration rate, *HbA1c* glycated hemoglobin, *SD* standard deviation.

**Figure 1:**
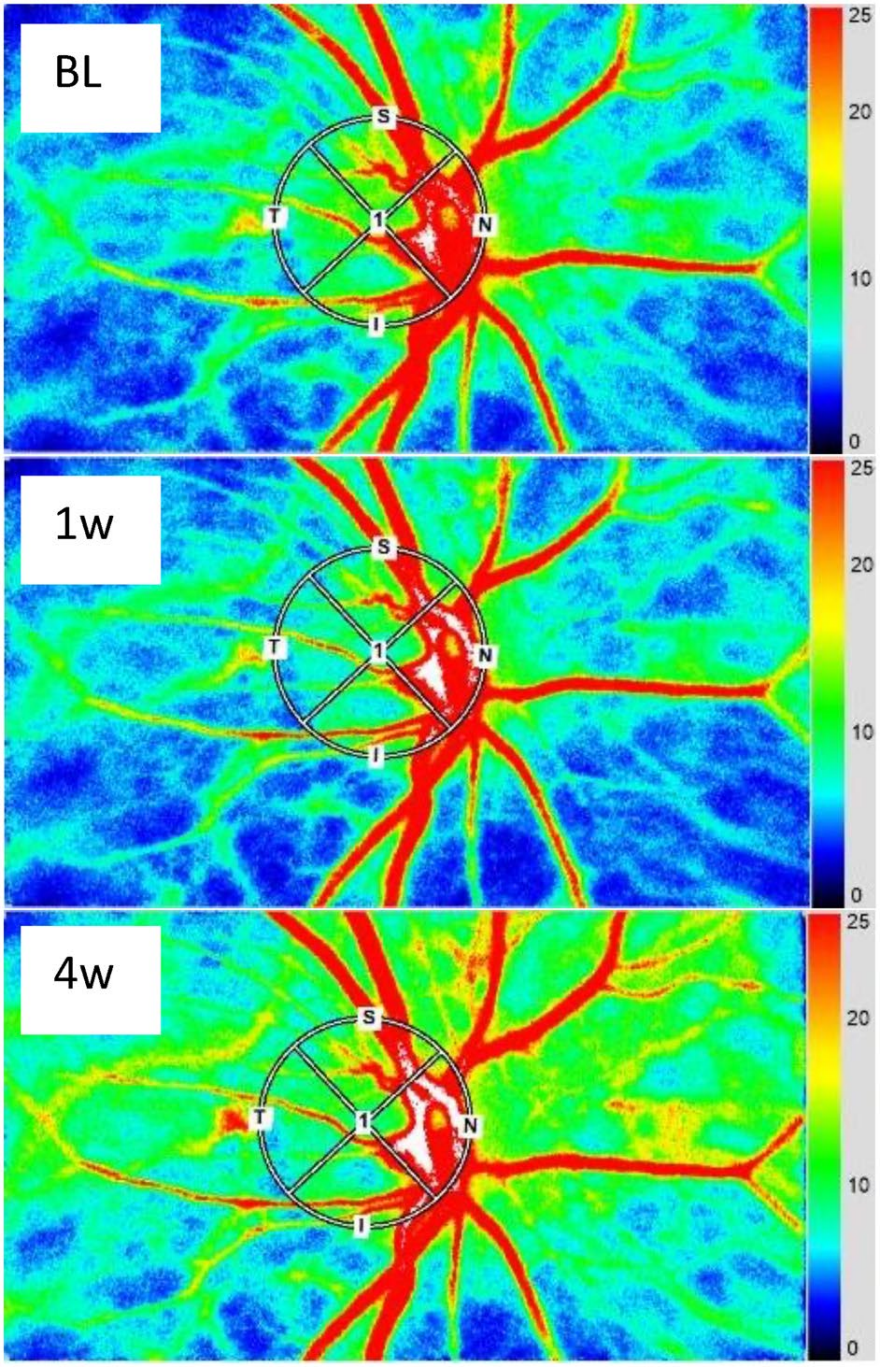
Representative color map images from laser speckle flowgraphy measurements of a patient from the current study. Measurements centered at the optic nerve head. *BL* baseline, *1w* image acquired 1 week after injection, *4w* image acquired 4 weeks after injection.

**Figure 2:**
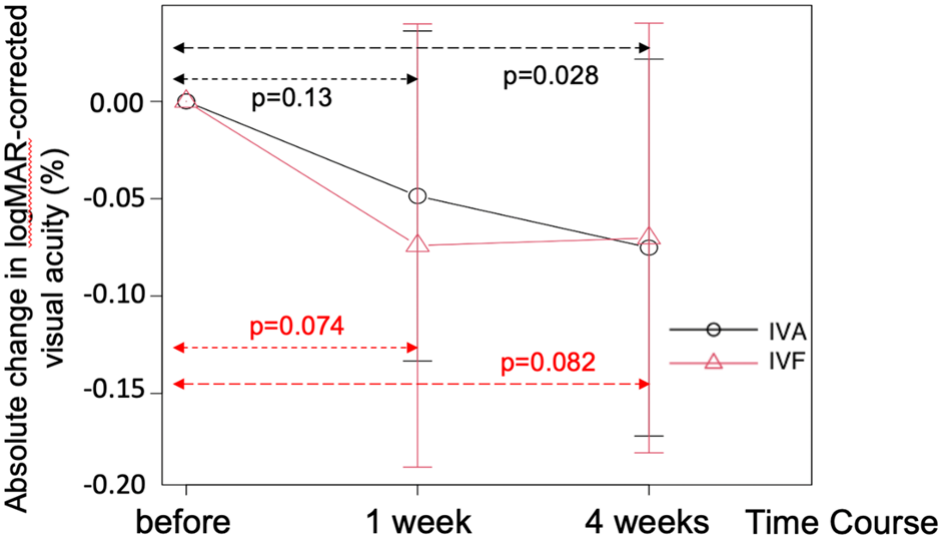
Absolute changes in the best-corrected visual acuity (logMAR) before, 1 week, and 4 weeks after IVA and IVF treatment. The changes in logMAR before and after injection. *IVA* intravitreal aflibercept, *IVF* intravitreal faricimab.

**Figure 3:**
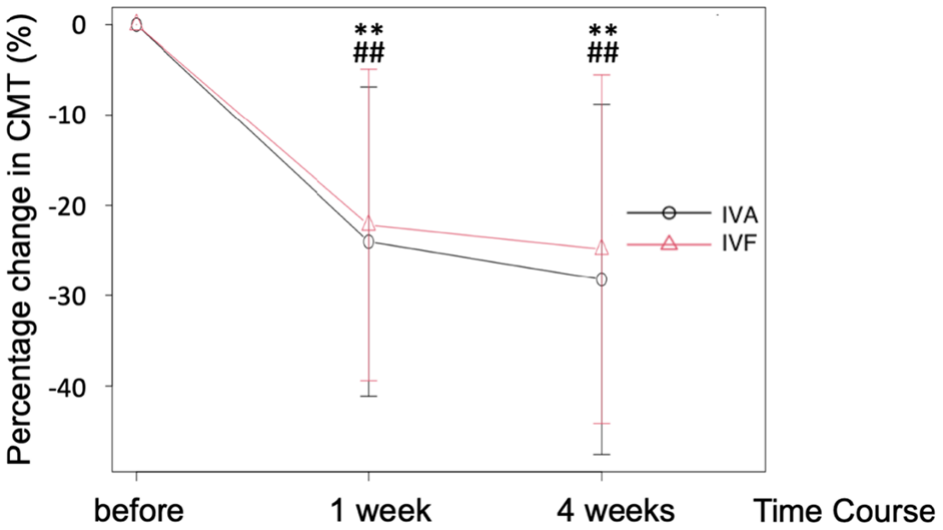
Percentage change in CMT before, 1 week, and 4 weeks after IVA and IVF treatment. % change in CMT before and after before injection. *CMT* central macular thickness, *IVA* intravitreal aflibercept, *IVF* intravitreal faricimab. **P < 0.01 vs before injection in IVA. ##P < 0.01 vs before injection in IVF.

**Figure 4:**
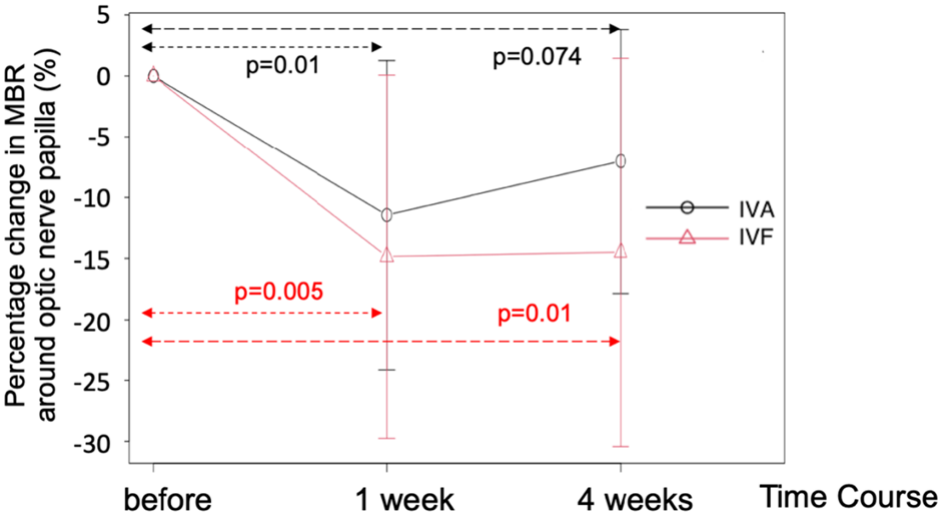
Percentage change in MBR around optic nerve disc before, 1 week and 4 weeks after IVA and IVF injection. % change in MBR before and after injection. *MBR* mean blur rate, *IVA* intravitreal aflibercept, *IVF* intravitreal faricimab.

Table 2 presents the absolute changes in best-corrected visual acuity (BCVA), CMT, MBR in all areas, systolic blood pressure (sBP), diastolic blood pressure (dBP), intraocular pressure (IOP), and ocular perfusion pressure (OPP). Figure 1 shows the representative color map images of the LSFG measurements of a patient in the present study. Figures 2–4 show the relative alterations from the baseline.

**Table 2:** Time courses of systemic and ocular parameters before, 1 week, and 4 weeks after injection.

	Before injection	1 week later	4 weeks later	*p*-value^#^ (two-way ANOVA)
**BCVA (logMAR, mean ± SD)**				
IVA	0.155 ± 0.147	0.097 ± 0.160	0.097 ± 0.135*	0.35
IVF	0.222 ± 0.178	0.155 ± 0.146	0.155 ± 0.182	
**CMT (μm, mean ± SD)**				
IVA	467 ± 136	346 ± 55**	327 ± 55**	0.69
IVF	463 ± 131	346 ± 76**	331 ± 64**	
**MBR (a.u., mean ± SD)**				
IVA	21.1 ± 5.4	18.0 ± 5.8*	19.5 ± 5.7	0.26
IVF	21.9 ± 6.6	17.2 ± 5.4**	16.4 ± 5.4*	
**Systolic BP (mmHg, mean ± SD)**				
IVA	138 ± 18	136 ± 20	137 ± 22	0.91
IVF	132 ± 20	134 ± 15	131 ± 21	
**Diastolic BP (mmHg, mean ± SD)**				
IVA	76 ± 11	79 ± 8	85 ± 12	0.38
IVF	80 ± 13	80 ± 12	83 ± 14	
**IOP (mmHg, mean ± SD)**				
IVA	13.3 ± 2.7	13.3 ± 3.5	13.3 ± 2.6	0.61
IVF	16.0 ± 2.6	13.7 ± 2.7	13.7 ± 3.8	
**OPP (mmHg, mean ± SD)**				
IVA	49.6 ± 6.8	52.6 ± 6.8	52.4 ± 8.6	0.50
IVF	48.6 ± 8.4	48.9 ± 7.9	53.0 ± 11.2	

*CMT* central macular thickness, *MBR* mean blur rate, *BCVA* best-corrected visual acuity, *BP* blood pressure, *IOP* intraocular pressure, *OPP* ocular perfusion pressure, *IVA* intravitreal aflibercept, *IVF* intravitreal faricimab, *SD* standard deviation.

*P < 0.05 vs before injection.

**P < 0.01 vs before injection.

#*p*-value examines the statistical differences between IVA and IVF by two-way analysis of variance.

No significant differences in sBP, dBP, IOP, or OPP were observed during the follow-up period (P > 0.05, one-way analysis of variance [ANOVA]). No significant differences were observed between IVA and IVF (P > 0.05, two-way ANOVA).

The logMAR visual acuity showed significant improvement during the follow-up period after IVA, whereas it tended to improve after IVF (IVA P = 0.01, IVF: P = 0.054, one-way ANOVA). The difference between the two drugs was not significant (two-way ANOVA, P = 0.732) (Fig. 2).

The mean CMT decreased significantly during the follow-up period. The CMT before, 1 week after, and 4 weeks after the administration of IVA were 467 ± 136 μm, 346 ± 55 μm, and 327 ± 55 μm, respectively (P < 0.001, one-way ANOVA). The CMT before, 1 week after, and 4 weeks after the administration of IVF were 463 ± 131 μm, 346 ± 76 μm, and 331 ± 64 μm, respectively (P < 0.001, one-way ANOVA). However, the difference between the two drugs was not significant (two-way ANOVA, P = 0.69) (Fig. 3).

The MBR in the disc area decreased significantly during the follow-up period following the administration of both drugs. The MBR before, 1 week after, and 4 weeks after the administration of IVA were 20.1 ± 5.4, 17.9 ± 5.8, and 18.7 ± 5.7, respectively (P = 0.0018, one-way ANOVA). The MBR before, 1 week after, and 4 weeks after the administration of IVF were 19.4 ± 6.6, 16.1 ± 5.4, and 16.1 ± 5.4, respectively (P = 0.0003, one-way ANOVA). No significant differences in the changes over time were observed between the two drugs (two-way ANOVA, P = 0.256). Comparison between the values at baseline and those at 4 weeks after treatment showed a significant decrease following the administration of IVF (P = 0.01, multiple comparison correction using the Bonferroni test); however, no significant decrease was observed after the administration of IVA (P = 0.074, multiple comparison correction using the Bonferroni test) (Fig. 4).

**Figure 5:**
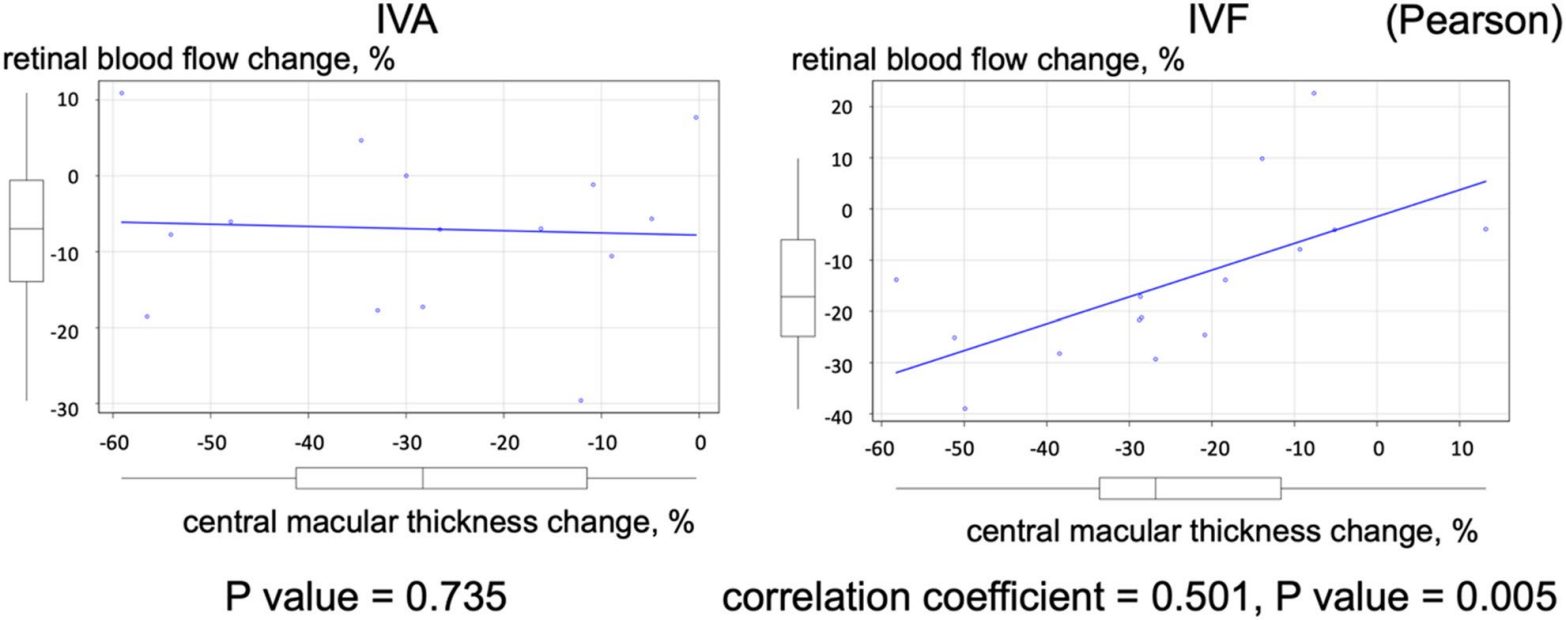
Correlation between changes in MBR and in CMT at 4 weeks post-injection. *CMT* central macular thickness, *MBR* mean blur rate.

The percentage change in the central foveal retinal thickness and MBR for each drug showed a significant association 4 weeks after the administration of IVF (correlation coefficient: 0.501, P = 0.005); however, no significant association was observed 4 weeks after the administration of IVA (P = 0.735) (Fig. 5).

## Discussion

IVF tended to improve the visual acuity in patients with treatment-resistant DME who switched from aflibercept to faricimab (P = 0.054, Fig. 2). Moreover, it reduced the CMT on optical coherence tomography (OCT) (Fig. 3) with the same efficacy as IVA. The retinal blood flow around the optic nerve head (ONH) also decreased 1 week after the injection in both trials. The blood flow remained significantly lower than that at baseline 4 weeks after the administration of IVF; however, this effect was not observed after the administration of IVA (Fig. 4). This is the first study to report the differences between the effects of IVA and IVF on retinal blood flow.

CMT on OCT decreased by > 20% from the baseline value 4 weeks after the administration of IVA and IVF in the same patient with DME, indicating that the effects of both drugs on macular edema are similar (Fig. 3). These results are consistent with those of the YOSEMITE and RHINE studies, which reported that the efficacy of faricimab was not inferior to that of aflibercept^2^. In addition, a subgroup analysis of patients in the YOSEMITE and RHINE trials revealed similar results in Asian and non-Asian countries^8^ and when limited to the patients in Japan^9^. Kusuhara et al. reported that the overall mean CMT after switching from IVA to IVF decreased significantly from the baseline value 1 month after administration^10^. The findings of the present study are consistent with those reported previously. Further studies with a longer follow-up period must be conducted to validate these findings, as these reports, including the YOSEMITE and RHINE studies, indicate that faricimab may be able to extend the durability of treatment for patients with DME.

BCVA tended to improve one month after the injection in both trials, and no significant difference was observed between the changes in BCVA reported by the trials (Fig. 2). These findings suggest that the potential of both drugs to improve visual acuity is almost identical. Moreover, these findings are comparable with those of the YOSEMITE and RHINE studies^2^.

Significant decreases in CMT were observed in both trials; however, no differences were observed between the changes in CMT reported by the trials (Fig. 3). The correlation between the changes in macular edema and visual acuity is not always linear following the intravitreal injection of ranibizumab in patients with DME^11^. Thus, a longer follow-up period is required to clarify the relationship between macular edema and visual acuity after the administration of IVF in patients with DME.

The present study revealed that the administration of IVF decreased the ocular blood flow in the disc in patients with DME and that the extent of this reduction was comparable with that induced by IVA (Fig. 4). This is the first study to measure the ocular blood flow before and after the administration of intravitreal faricimab. VEGF has been shown to induce vasodilation in porcine arterioles^12^ and increase the retinal blood flow^13^ through the production of nitric oxide from the vascular endothelium. Thus, it is reasonable to assume that the retinal blood flow was reduced following the intravitreal injection of anti-VEGF drugs, as reported by several previous clinical studies. However, it remains unclear whether angiopoietin-2 plays a role in the regulation of blood flow in the retina. The present study demonstrated that the extent of blood flow reduction following the administration of IVA and IVF was similar, indicating that these reductions in blood flow were primarily induced by the reduction in VEGF in the eye at least 1 week after the injection. The significant reduction in retinal blood flow was maintained 4 weeks after the administration of IVF; however, the retinal blood flow returned to baseline 4 weeks after the administration of IVA (Fig. 4). Although the number of patients included in the present study was small and the follow-up duration was short, the findings indicate that faricimab may yield a sustained reduction of ocular blood flow 4 weeks after treatment, unlike IVA. Thus, faricimab can be used for the treatment of macular edema in clinical practice if the reduction in the ocular blood flow is maintained.

The present study revealed a significant negative correlation between the changes in ocular blood flow and CMT 4 weeks after administering IVF (Fig. 5); however, no significant correlation was observed 4 weeks after administering IVA. Although the mechanism underlying this correlation is difficult to explain, the reduction in ocular blood flow after administering IVF may be a positive predictor for evaluating whether the drug is appropriate for use in patients with DME. Unfortunately, no correlations were observed between the changes in visual acuity and CMT or blood flow in the present study. Further long-term clinical studies must be conducted to determine whether the reduction in blood flow can be used to predict the recurrence of DME, as the recovery of visual acuity requires a longer duration after anti-VEGF treatment.

Sabaner et al. demonstrated the constriction of retinal arterioles in treatment-naïve patients with DME following the administration of three consecutive monthly doses of 2 mg IVA. A semi-automatic computer-based software program was used to calculate the retinal vessel diameter in their study^6^. Although blood velocity and blood flow were not measured in their study, the constriction of the retinal arterioles may have reduced the retinal blood flow, which is comparable with the findings of the present study. Mizui et al. reported that intravitreal injection of ranibizumab reduced retinal blood flow in treatment-naïve patients with DME using LSG^4^. They also reported that the reduction in retinal blood flow was correlated with the regression of the morphological pathology, which is comparable with the findings of the present study. This is the first study to evaluate the effects of faricimab on ocular blood flow in patients with DME. The strength of this study is that it compared the changes in ocular blood flow after IVA and IVF in the same patients who were resistant to IVA.

It is known that retinal laser photocoagulation has an impact on retinal and choroidal blood flow and in patients with diabetes^14,15^. Although some patients had previously undergone photocoagulation in the current study, all had undergone retina laser photocoagulation at least 1 year prior to IVA administration and had no new photocoagulation during anti-VEGF treatment follow-up. Therefore, we believe that the previous photocoagulation may have little influences our results.

Although the effect of Ang-2 on retinal blood flow was not evaluated in this study, Ang-2 release from endothelial cells with reduced nitric oxide bioavailability has been previously postulated to contribute to endothelial dysfunction in patients with severe malaria^16^. Thus, the reduction in Ang-2 by IVF injection may improve the vascular endothelial function and reduce VEGF. Because VEGF can induce the production of nitric oxide from the retinal vascular endothelium^12^, its decrease may reduce the release of nitric oxide, resulting in a persistent decline in retinal blood flow at 4 weeks following the injection.

The present study has some limitations. First, this was a retrospective study with a short follow-up period and a small sample size. Second, the number of injections administered before the final IVA injection was unclear. Third, the duration of DME was also unclear. Fourth, the effect of the systemic parameters on the blood flow data was unclear. For instance, renal function may affect retinal blood flow in patients with diabetes. Fifth, differences in the molecular weight and structure of the drugs may have influenced our results. Especially, in the faricimab molecule, an Fc fragment has been modified to reduce systemic exposure time and associated side effects by reducing immune system stimulation^17^.

Lastly, the effect of IVF on the ocular blood flow in patients with DME resistant to aflibercept was examined in this study; therefore, further studies with treatment-naïve patients must be conducted to examine the effect of IVF on ocular blood flow after excluding confounding factors.

In conclusion, our findings suggest that IVF may be comparable to IVA regarding the effects on visual function and macular edema with prolonged reduction of retinal blood flow, which may be related with the reduction of macular edema in patients with DME. Further long-term follow-up studies must be conducted to determine the effects of faricimab on ocular blood flow in patients with DME.

## Methods

The medical records of patients with DME who visited the department of ophthalmology, Nihon University Itabashi Hospital, between October 1, 2022, and January 31, 2023, were retrospectively analyzed. Patients who received IVF injections initially owing to resistance to aflibercept at least three months prior were included in the analysis. Macular edema was defined as a CMT of ≥ 300 μm on OCT with intraretinal or subretinal fluid. In the present study, “treatment resistance” was defined as improvement in retinal thickness by less than 10% at 3 months after the last IVA injection.

Among the patients with DME who received multiple doses of intravitreal anti-VEGF injections, the last dose of aflibercept and the first dose of faricimab were included in the analysis. Among the patients who received vitreous injections in both eyes, the eye in which faricimab was administered first was included in the analysis. We excluded from this study the patients with the history of ocular surgeries, such as vitrectomy or glaucoma surgery.

The primary endpoints were the changes in BCVA, CMT on OCT, and MBR of the optic nerve papillary blood flow on LSFG before the administration and 1 and 4 weeks after the administration of the last aflibercept dose and the first faricimab dose. In addition, the changes in IOP, OPP, sBP, and dBP were also evaluated. Patients with abnormal examination data owing to the presence of other ophthalmologic diseases, such as corneal erosion, and those with missing data were excluded.

LSFG-NAVI (SoftCare Co., LTD, Fukutsu, Japan) and Mirante (NIDEK Co., LTD, Gamagori, Japan) were used to perform the LSFG and OCT examinations, respectively. The MBR on LSFG in the present study was calculated for the total ONH area within an ellipsoid region of interest using LSFG analyzer software (V3.8.0.4). This value was referred to as the MBR of all disc areas (Fig. 1). A circle was set around the ONH to evaluate the blood flow within. A total of 118 MBR images were acquired from the circular area within a 4 s period tuned to the cardiac cycle. The pulse waves of the changing MBR values, which corresponded to each cardiac cycle, were displayed on the analysis screen. The results (normalized to one pulse) were displayed subsequently, and the pulse waveform and MBRs were analyzed on this screen. The MBR was determined using the mean MBR during one normalized cardiac cycle^18^.

All statistical analyses were performed using EZR Ver 1.60, statistical software that combines the capabilities of R and R Commander (Saitama Medical Center, Jichi Medical University, Saitama, Japan)^19^. EZR is now being distributed on the following website: http://www.jichi.ac.jp/saitama-sct/. The Kolmogorov–Smirnov test was used to confirm that sample normality was not rejected. The Mauchly test was used to confirm that sphericity was not rejected. The Greenhouse–Geisser correction formula was used if sphericity was rejected. One-way repeated-measures ANOVA was performed to analyze the changes over time before and after each administration. Multiple comparisons were performed using Bonferroni’s method if a significant difference was observed in the one-way repeated-measures ANOVA. Two-way repeated-measures ANOVA was used to examine whether a significant difference was observed between IVA and IVF. The percentage changes in CMT on OCT, MBR of the optic nerve blood flow on LSFG, IOP, OPP, sBP, and dBP compared with the baseline values were determined. In addition, the absolute change in the corrected visual acuity was also assessed. Pearson product-moment correlation coefficient was used to evaluate the correlation between the changes in MBR and CMT.

This study protocol was approved by the ethics committee of Nihon University (RK-230314-9), and all procedures were conducted in accordance with the tenets of the Declaration of Helsinki. Informed consent was waived by the ethics committee of Nihon University because this was a retrospective study, and all patient data were protected and kept confidential by the researchers. Additionally, we provided opt-out documentation to give the participant a chance to refuse.

## Data Availability

Raw data were generated at the department of ophthalmology, Nihon University Itabashi Hospital. Derived data supporting the findings of this study are available from the corresponding author T.N. on reasonable request.
