# Author Correction: Effects of switching from intravitreal injection of aflibercept to faricimab on ocular blood flow in patients with diabetic macular edema

**DOI:** 10.1038/s41598-025-03157-7

**Published:** 2025-06-02

**Authors:** Yoshinari Saima, Harumasa Yokota, Akifumi Kushiyama, Junya Hanaguri, Akira Ohno, Koyo Takase, Ruri Sugiyama, Kimimasa Muranaka, Satoru Yamagami, Taiji Nagaoka

**Affiliations:** 1 Division of Ophthalmology, Department of Visual Sciences, Nihon University School of Medicine, Tokyo, Japan; 2 Department of Ophthalmology, Saitama Medical Center, Jichi Medical University, Saitama, Japan; 3 Department of Pharmacotherapy, Meiji Pharmaceutical University, Tokyo, Japan; 4 Tokiwadai Muranaka Eye Clinic, Tokyo, Japan; 5 Department of Ophthalmology, Asahikawa Medical University, Asahikawa, Hokkaido Japan

Correction to: *Scientific Reports* 10.1038/s41598-024-63435-8, published online 14 June 2024

The original version of this Article contained an error in the Methods section, where the Intravitreal Aflibercept Injection (IVA) was incorrectly given as Intravitreal Faricimab (IVF).

As a result,

‘In the present study, “treatment resistance” was defined as improvement in retinal thickness by less than 10% at 3 months after the last IVF injection.’

now reads,

‘In the present study, “treatment resistance” was defined as improvement in retinal thickness by less than 10% at 3 months after the last IVA injection.’

The original Article has been corrected.

